# Domestication drive the changes of immune and digestive system of Eurasian perch (*Perca fluviatilis*)

**DOI:** 10.1371/journal.pone.0172903

**Published:** 2017-03-03

**Authors:** Xiaowen Chen, Jun Wang, Long Qian, Sarah Gaughan, Wei Xiang, Tao Ai, Zhenming Fan, Chenghui Wang

**Affiliations:** 1 Key Laboratory of Freshwater Fisheries Germplasm Resources, Ministry of Agriculture, Shanghai Ocean University, Shanghai, China; 2 Fisheries Technology Extension Station, Xinjiang Production and Construction Corps, Urumqi, Xinjiang, China; 3 Department of Biology, University of Nebraska at Omaha, Omaha, Nebraska, United States of America; Xiamen University, CHINA

## Abstract

Domestication has altered a variety of traits within the Eurasian perch (*Perca fluviatilis*), including phenotypic, physiological and behavioral traits of Eurasian perch (*Perca fluviatilis*). Little is known, however, about the genetic changes between domesticated and wild Eurasian perch. In this study, we assembled a high-quality *de novo* reference transcriptome and identified differentially expressed genes between wild and domesticated Eurasian perch. A total of 113,709 transcripts were assembled, and 58,380 transcripts were annotated. Transcriptomic comparison revealed 630 differentially expressed genes between domesticated and wild Eurasian perch. Within domesticated Eurasian perch there were 412 genes that were up-regulated including *MHCI*, *MHCII*, *chia*, *ighm* within immune system development. There were 218 genes including *try1*, *ctrl*, *ctrb*, *cela3b*, *cpa1* and *cpb1*, which were down-regulated that were associated with digestive processes. Our results indicated domestication drives the changes of immune and digestive system of Eurasian perch. Our study not only provide valuable genetic resources for further studies in Eurasian perch, but also provide novel insights into the genetic basis of physiological changes in Eurasian perch during domestication process.

## Introduction

Domestication is the process of manipulating specific attributes of a species’ in an effort to maximize agricultural and commercial ventures, such as the pet trade, or conservation efforts by controlling a species’ movement, feeding and habitats [1]. Humans have played a vital role in the domestication process for many economically valuable and endangered species for approximately 11,000 years [1, 2]. Selective breeding during the long term domestication causes a series of phenotypic changes that may be evident in the morphology, physiology, behavior and reproduction of a species [3, 4]. The study of domesticated species has led to increased interest in several important issues in genetics and evolutionary biology, including the underlying genetic architecture of local adaptations and parallel evolution [5]. Understanding the process of animal domestication and its reciprocal impacts on humans and animal domesticates is critical for human life, conservation and evolutionary biology [1, 3, 5].

Many aquatic species were domesticated from their wild ancestors for the benefit of humans. Aquaculture is a form of domestication which has been considered as a profitable venture. Fish harvested through aquaculture efforts were approximately 73.8 million tons in 2014 from a total of 580 species or species groups farmed around the world. These harvests yield an estimated first-sale value of US$160.2 billion [6]. Aquaculture efforts may be particularly effective and convenient solution if natural population of many species declined dramatically [7]. The commercial profitability and potential sustainability has resulted in large scale domestication efforts for many aquatic species.

Eurasian perch (*Perca fluviatilis*) is a common predatory freshwater fish species and an economical aquaculture species [8–10]. It is widely distributed across Europe, and is also found in the Irtse (Eltrixhe) River, located in the northern part of Xinjiang Province [11]. Eurasian perch is a promising aquaculture candidate due to high demand for the excellent fillet. Due to the large human consumption and the decline of wild Eurasian perch population, domestication of Eurasian perch was conducted in the early 1990 [12]. The intensive production of Eurasian perch can be sustained within controlled systems throughout the lifecycle of Eurasian perch and without wild input [7, 12]. The process of domestication changed the morphology, physiology, and reproduction of the domesticated Eurasian perch. Domesticated Eurasian perch demonstrate significant differences in growth performance [13, 14], immune capacity and resistance to chronic stress [8, 12, 15] as well as differences in the values of n-3 polyunsaturated fatty acid in the liver [16], the gonadosomatic (GSI), hepatosomatic (HSI), and viscerosomatic (VSI) indices [17], which facilitate being reared in limited spaces at high densities under commercially prepared diets [7, 18]. Following spawning however, domesticated Eurasian perch demonstrate a higher broodstock mortality [17]. Besides, the domesticated and wild Eurasian perch have some differences in the texture and flesh color [19–21].

This domestication process has caused aquatic species, such as Eurasian perch to develop a set of adapted traits, “domesticated traits”, such as high prolificacy, resistance to disease, and rapid growth rate [7, 18]. Phenotypic comparison revealed vital commercial trait differences between domesticated and wild type fish including reproduction, immunology, skin color, diet change, etc [22–24]. Although many phenotypic variations have been identified, the molecular basis for these “domesticated traits” have not been well researched. Understanding the underlying mechanisms of these phenotypic preferences may facilitate future commercial ventures.

RNA-Seq is an effective technology to explore different gene expression profiles and has been widely used for domestication studies [2, 25–29]. In this study, we assembled a comprehensive *de novo* transcriptome of the Eurasian perch in order to investigate the phenotypic and genetic changes during domestication process of Eurasian perch and to identify candidate genes and relevant metabolic pathways associated with the changes during domestication process.

## Materials and methods

### Animal sampling and ethics

This study was approved by the Institutional Animal Care and Use Committee (IACUS) of Shanghai Ocean University (Shanghai, China). Sampling procedures complied with the guidelines of the IACUS on the care and use of animals for scientific purposes. Two groups (wild and domesticated) of fish were collected within the Xin Jiang Province of China. Wild individuals were sampled from the Eltse River and domesticated fish individuals from the fifth generation (F5) of closed selective line for growth traits were collected from the Fisheries Technology Extension Station of Xinjiang Production and Construction Corps (Urumqi, Xinjiang). Two biological replicates were collected for wild and domesticated groups. Following anaesthetization (in tricaine methanesulfonate (MS222) at a concentration of 1:18000), each individual was weighed, and then fresh brain, heart, gill, liver, muscle, kidney, and pancreas tissues were quickly collected and immediately stored in liquid nitrogen for RNA isolation. After RNA extraction, RNA from each tissue type were diluted to equal concentrations and then pooled together for each fish individual for RNA-Seq sequencing.

### RNA isolation and RNA-seq library preparation

Total RNA was extracted from approximately 80 mg of tissues from each specimen with TRIzol Reagent (Tiangen, China) according to the manufacturer’s instructions. Extracted RNA was purified using the RNA clean Kit (Tiangen, China). The RNA integrity and quantity were examined using agarose gel electrophoresis and an Agilent 2100 Bioanalyzer (Agilent, Shanghai, China), respectively. A total of 5 μg RNA with an RNA integrity exceeding 8.0 was used for RNA-seq library construction using the Truseq^™^ RNA sample Prep Kit for Illumina (Illumina, USA). These indexed libraries were sequenced on an Illumina Hiseq^™^4000, which produced 150 bp pair-end reads.

### Cleaning and assembly of RNA sequencing reads

Raw reads were first quality-filtered using the Trimmomatic read trimming tool (for detailed methodology please refer to our previous study) [30]. All cleaned reads were used for the reference transcriptome assembly based on Trinity version 2.0.6 with the paired-end mode, and a minimum assembled contig length of 300bp [31]. Redundant transcripts were removed from the transcriptome by CD-Hit software under default parameters [32]. Finally, BWA-0.17 software was used to map the raw to the assembled transcriptome to determine the extent that the RNA-Seq reads representated the assembly [33]. Mapping statistics were then estimated by the flagstat command implemented in SAMtools-0.18 software [34].

### Transcriptome annotation

The assembled reference transcriptome was annotated by BLASTX against the NCBI-NR and UniProt protein databases, with a cutoff E-value smaller than 1E-5. Functional Gene Ontology (GO) assignment and Kyoto Encyclopedia of Genes and Genomes (KEGG) pathways annotation were generated through BLAST2GO 3.0 software based on the BLASTX results of NCBI-NR database [35]. The protein-coding DNA sequence region (CDS) was predicted using TransDecoder 2.0.1 implemented in the Trinity software. Sequences with a corresponding protein length greater than 100 were retained for further analysis. Protein sequences were queried against the PFAM-A database using the HMMER software with the hmmscan command to predict functional protein domains [36]. BUSCO software was used to evaluate comprehensiveness of the transcriptome’s assembly and annotation using vertebrates as a reference database [37].

### Differential expression analysis

Cleaned raw reads were mapped to the assembled reference transcriptome using Bowtie 1.0.0 [38]. Transcripts abundance was estimated using the RSEM software and the FPKM (fragments per kilobase per transcript per million mapped reads). Transcripts were defined as expressed if they had an FPKM > 0.5 in both replicates [39]. The resulting data matrix was generated by “rsem-generate-data-matrix” script and contains the expression value (FPKM) for all samples of both the wild and domesticated groups. The false discovery rate (FDR) of differentially expressed genes (DEGs) was calculated by edgeR 2.14 using *P* < 0.001 for [40]. After normalizing the DEGs FPKM values using log2 and centering them around the median, cluster analysis was performed using the hierarchical cluster method based on the Euclidean distance using a Trinity Perl script [31]. GO enrichment analysis of the DEGs was conducted by Blast2GO software with the Fisher algorithm (*P*<0.05) [35].

### SNPs calling

Raw reads from wild and domesticated groups were mapped to the reference transcriptome using bwa (v 0.7.9a-r786) with the bwa-mem algorithm [33]. Potential SNPs were called using SAMtools (v 0.1.18) software [34]. Potential SNPs for wild and domesticated perch individuals were filtered according to the following stringent criteria: read depths >10, and a minimum quality score of 20 in each group.

### RT-PCR

In order to validate the RNA-seq differential gene expression analysis, quantitative real-time PCR (qRT-PCR) was carried out on specific immune (pancreas) and digestive (intestines) organs between domesticated and wild Eurasian perch. Eight genes related to immunity and eight genes related to digestive biological processes were chosen for qRT-PCR assays. PCR primers were designed based on the assembled transcriptome sequences (S1 Table). *β-actin* was used as an internal reference gene to normalize the gene expression level. qRT-PCR was conducted using SYBR Green Premix Ex Taq (Takara, Japan) in a CFX96 real-time PCR system (Bio-Rad, USA). A standard curve was generated to assess accuracy, and primers with an amplification efficiency between 95% and 105% were chosen for qRT-PCR. Three biological and four technical replicates were used for each gene. qRT-PCR was performed in a 25 μ l reaction mixture including 12.5 μ l SYBR Premix Ex Taq^™^ II (2×), 1 μ l each primer (10 μ M), 2 μ l cDNA, and 8.5 μ l ddH2O. The PCR procedure was as follows: 95°C for 30 s, followed by 40 cycles of 95°C for 5 s and 60°C for 30 s, with a 0.5°C/5 s incremental increase from 60°C to 95°C, 30 s elapse time for each cycle. The relative expression was estimated using the 2^–ΔΔCt^ method with domesticated individuals as a calibration control [41]. Relative expression results were presented as the fold-change relative to domesticated individuals. Statistical significance (*P* < 0.05) was determined using one-way ANOVA and Duncan’s multiple range tests under SPSS 17.0.

## Results

### Reference transcriptome assembly and annotation

**Fig 1:**
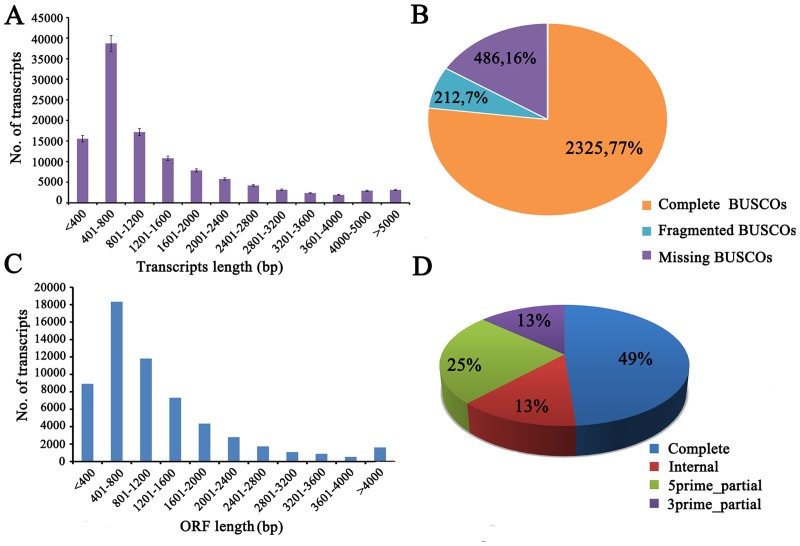
Assessment of Eurasian perch transcriptome assembly and annotation. (A). Histogram of transcripts length distribution. (B). Evaluation of the transcriptome assembly based on vertebrate data set through BUSCO software. (C). Histogram of ORF length distribution. (D). Statistics on ORF completeness.

Sequencing generated a total of 110,491,446 and 93,901,694 paired-end reads of 150 bp in length for the domesticated and wild groups respectively (SRX2317605). Trimming low quality reads left 83,595,948 and 71,419,870 clean paired-end reads for the domesticated and wild group, respectively. Approximately 23 Gb (giga base) of cleaned reads were used for the *de novo* transcriptome assembly. After removing redundant transcripts, 113,709 assembled transcripts were obtained, with an N50 and mean length of 2,142 bp and 1,373 bp, respectively (Table 1 and Fig 1A). A high proportion of reads,98.54% (89.73% properly paired) reads were able to be mapped back to the reference transcriptome, indicating that a high quality reference transcriptome assembly (S2 Table).

**Table 1:** Summary statistics of Eurasian perch transcriptome.

**Sequencing**	
Raw reads (pair-end)	204,393,140
Clean reads (pair-end)	155,015,818
Total nucleotides (bp)	2.3×10^10^
**Assembly**	
No. of transcripts	113,709
No. of unigenes	83,834
No. of predicted CDS	51,869
No. of N50 transcripts	21,355
N50 length	2,142
Mean transcripts length	1,373
**Annotation**	
NCBI-NR	58,380
UniProt	51,771
GO	26,598
KEGG	22,971

A total of 58,380 and 51,771 transcripts could be annotated by BLASTX searches against NCBI-NR and Uniprot protein databases, corresponding to 19,872 Uniprot proteins (Table 1). In which, 10,592 (53.3%) of the Uniprot proteins could be represented by nearly full-length assembled transcripts with greater than 80% alignment coverage (S3 Table), suggesting that half of the assembled transcripts were full-length or nearly full length. Using the BUSCO software, 2,325 completely conserved orthologs were identified from the vertebrate database (Fig 1B). Species distribution of top BLAST hits showed Eurasian perch reference transcriptomic sequences had the largest number of homologous sequences in *Larimichthys crocea*, which is considered the closest relative to perch with an assembled genome (S1 Fig) [42]. TransDecoder prediction analysis demonstrated that nearly 50% of the 51,869 predicted protein coding sequences (CDS) possessed an average nucleic acid length of 1,206bp containing complete open reading frames (ORFs) (Fig 1C and 1D).

Using Blast2GO software, 26,598 transcripts (23.39%) were mapped to the following three GO categories: biological processes, molecular functions and cellular components (S2 Fig). A large number of transcripts were assigned to a wide range of gene ontology categories. In biological processes, most of the GO-mapped transcripts were related to cellular, metabolic and single-organism processes, suggesting a high degree of metabolic activity of Eurasian perch. We identified 6,065, 4,161, 2,122 and 584 transcripts involved in biological regulations (GO:0065007), response to stimulus (GO: GO:0050896), developmental process (GO: 0032502), and immune system process (GO: 0002376), respectively. Binding (GO:0005488) was the most represented molecular function category. Cell (GO:0005623), membrane (GO:0016020) and organelle (GO:0043226) were the most represented GO terms for the cellular component category. A total of 22,971 transcripts were annotated with corresponding enzymes, and 120 KEGG pathways were identified through functional analysis of the Eurasian perch (S4 Table). 50,440 protein sequences were predicted to have functional protein domains through HMMER software using the PFAM-A database (S5 Table).

### Differentially expressed genes

**Fig 2:**
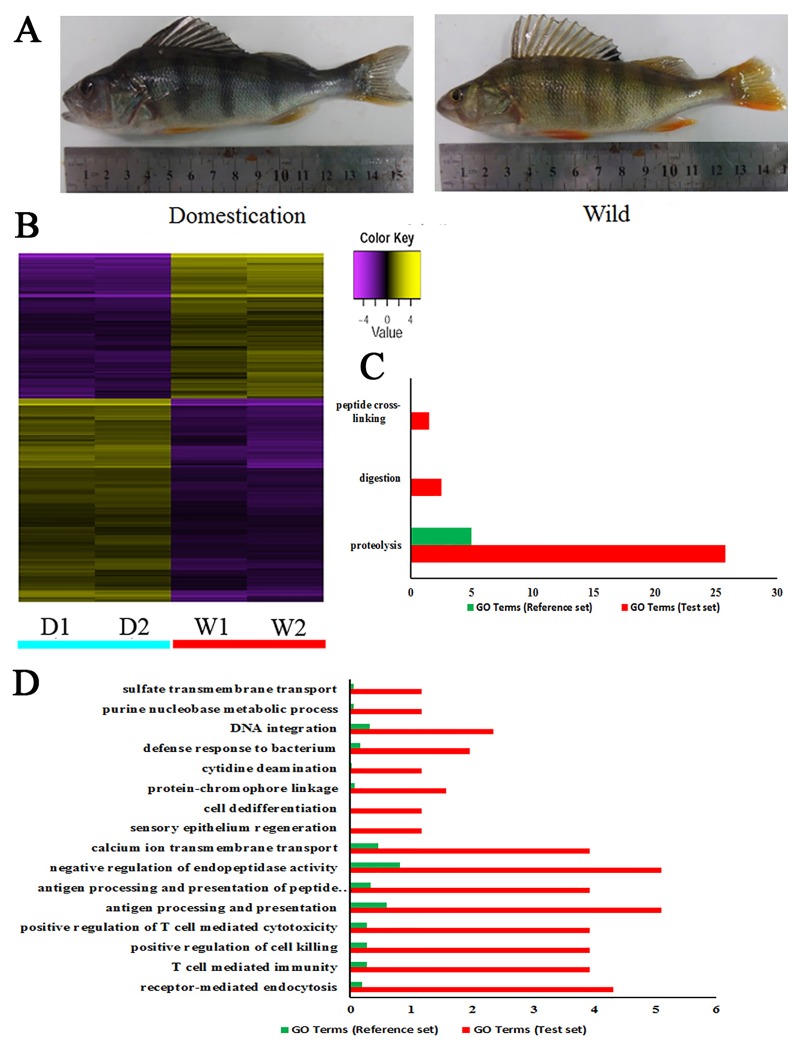
Heat map and GO enrichment of differentially expressed genes between domesticated and wild Eurasian perch. (A). digital photograph of domesticated and wild Eurasian perch. (B). Heat map of differentially expressed genes, “D” indicates domesticated perch and “W” indicates wild perch. (C). GO enrichment analysis of down-regulated genes in domesticated perch, with whole transcriptome genes as reference set and down-regulated genes as a test set. (D). GO enrichment analysis of up-regulated genes in domesticated perch, with whole transcriptome genes as reference set and up-regulated genes as a test set.

**Fig 3:**
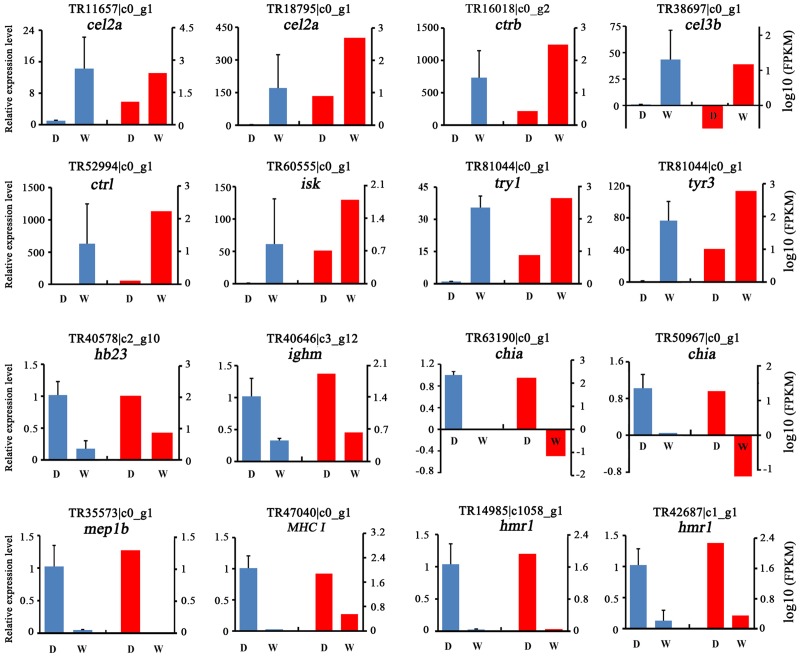
Expression profiles of 16 differentially expressed genes from RNA-Seq (red) and qRT-PCR (blue) between domesticated and wild perch. “D” indicates domesticated perch and “W” indicates wild perch.

**Fig 4:**
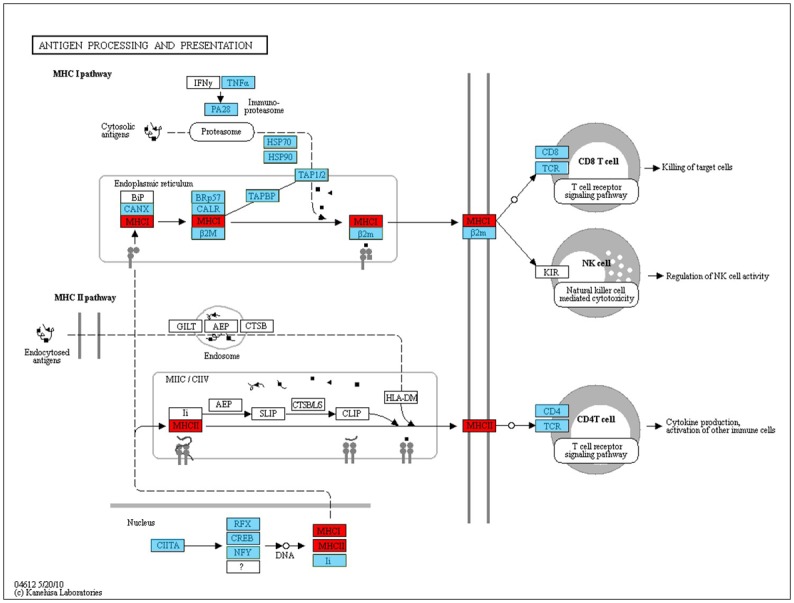
Antigen processing and presentation pathway of Eurasian perch transcriptome. Genes in blue boxes indicated these genes were identified and annotated in Eurasian perch transcriptome. Genes in red boxes indicated up-regulated genes in domesticated perch.

A total of 630 annotated unigenes were differentially expressed between the domesticated and wild groups (*P*<0.001, fold change >2^2). Amongst these annotated unignes, 218 were up-regulated and 412 unigenes were down-regulated in the domesticated group compared to wild group (Fig 2A and 2B). Genes which were highly expressed in the domesticated group were mostly enriched in immune system development (Fig 2C). For instance, the genes of MHC class I antigen (*MHCI*), MHC class II antigen (*MHCII*), L-amino-acid oxidase, Acidic mammalian chitinase (*chia*), and Immunoglobulin mu heavy chain (*ighm*) were enriched in T cell mediated immunity (GO:0002456), positive regulation of T cell mediated cytotoxicity (GO:0001916), antigen processing and presentation of peptide antigen via MHC class I (GO:0002474), and defense response to bacterium (GO:0042742) (Figs 2 and 4). Genes which had a lower expression in the domesticated group were mostly enriched in proteolysis (GO:0006508) and digestion (GO:0007586) (Fig 2D). Genes such as Trypsin-1 (*try1*), Chymotrypsin-like protease CTRL-1 (*ctrl*), Chymotrypsin B (*ctrb*), Chymotrypsin-like elastase family member 3B (*cel3b*) Carboxypeptidase A1 (*cpa1*), and Carboxypeptidase B (*cpb1*) were associated with digestive processes (Figs 2 and 3).

### RT-PCR validation

Sixteen genes from DEGs were selected for verification by qRT-PCR analysis. Among these, eight genes were associated with digestive processes (*cel2a*, *ctrb*, *cel3b*, *ctrl*, *isk*, *tyr1*, *tyr3*) and showed higher expression levels in wild Eurasian perch (Fig 3). Another eight genes (*hb23*, *ighm*, *chia*, *mep1b*, *Mhc1*, *hmr1*) showed higher expression levels in domesticated Eurasian perch, which indicating a higher immune response. These results indicated that genes which selected for verification were highly correlated with the DEGs results and confirmed the GO enrichment results (Figs 2 and 3).

### SNP calling

We identified 35,622 SNPs in 16,403 transcripts under stringent criteria that can be used to distinguish wild and domesticated perch. 3,741 putative homozygous SNPs were discovered, including 2,275 potential SNPs in the domesticated group showing the reference nucleotide, while the wild group showing the variant nucleotide, and remaining 1,466 potential SNPs with the opposite (S6 Table). A total of 31,881 heterozygous SNPs were identified (S6 Table). Additionally, 91, 351, and 691 putative SNPs were identified and associated with the following GO biological processes: growth (GO:0040007), immune system (GO:0002376), and response to stimulus (GO:0050896), respectively (Table 2 and S7 Table, S3 Fig).

**Table 2:** Putative SNP markers differentiating domesticated and wild perch with the associated GO process. The appropriate GO process is distinguished as follows: growth (GO:0040007), immune system process (GO:0002376), and response to stimulus (GO:0050896) from the reference transcriptome*.

Transcripts ID	Position	Domesticated/Wild GT^a^	Domesticated/wild DP^b^	Associate gene name
Growth (GO:0040007)				
**TR12134|c0_g3_i1**	300	GA/GG	40/26	cdc42 homolog
**TR19596|c0_g2_i1**	460	TT/GG	763/1151	dual specificity protein phosphatase 6
**TR19596|c0_g2_i1**	472	AA/GG	738/1115	dual specificity protein phosphatase 6
**TR19596|c0_g2_i1**	1378	TT/AA	590/372	dual specificity protein phosphatase 6
**TR19596|c0_g2_i1**	2550	GG/AA	277/127	dual specificity protein phosphatase 6
**TR19596|c0_g2_i1**	838	AA/GG	143/210	dual specificity protein phosphatase 6
**TR20791|c0_g1_i2**	2544	AG/AA	37/21	cell division control protein 42 homolog isoform x2
**TR23201|c0_g9_i1**	1225	TC/TT	23/17	beta-galactoside alpha- -sialyltransferase 2
**TR26783|c2_g1_i1**	1175	CT/CC	184/305	bone morphogenetic protein 6
**TR26783|c2_g1_i1**	1157	GC/GG	176/305	bone morphogenetic protein 6
**TR26783|c2_g1_i1**	912	TG/TT	109/186	bone morphogenetic protein 6
**TR26783|c2_g1_i1**	795	GA/GG	92/174	bone morphogenetic protein 6
**TR26783|c2_g1_i1**	971	CC/AA	66/140	bone morphogenetic protein 6
**TR26783|c2_g1_i1**	982	CC/TT	58/136	bone morphogenetic protein 6
**TR26783|c2_g1_i1**	2463	AA/GG	55/41	bone morphogenetic protein 6
**TR26783|c2_g1_i1**	3955	CG/CC	43/20	bone morphogenetic protein 6
**TR2947|c0_g1_i1**	1055	AA/TT	112/139	endothelial cell-specific molecule 1
**TR2947|c0_g1_i1**	1057	TT/AA	110/136	endothelial cell-specific molecule 1
**TR2947|c0_g1_i1**	1140	CT/CC	46/78	endothelial cell-specific molecule 1
Immune system process (GO:0002376)				
**TR10178|c0_g1_i1**	2988	GG/GA	11/11	cystic fibrosis transmembrane conductance regulator
**TR12134|c0_g3_i1**	300	GA/GG	40/26	cdc42 homolog
**TR12177|c0_g1_i1**	642	CG/CC	432/285	cd59 glyco
**TR12177|c0_g1_i1**	612	AA/GG	854/381	cd59 glyco
**TR12177|c0_g1_i1**	213	GG/GA	2541/1281	cd59 glyco
**TR12177|c0_g1_i1**	309	GT/GG	3550/1779	cd59 glyco
**TR13075|c0_g1_i1**	425	TT/GT	40/42	ap-2 complex subunit mu isoform x1
**TR14727|c0_g1_i3**	1253	GG/GT	23/29	granulocyte colony-stimulating factor receptor
**TR14985|c1041_g1_i1**	330	CT/CC	1536/1221	calmodulin
**TR15597|c0_g2_i1**	83	GG/GA	67/22	mhc class i partial
**TR15597|c0_g2_i1**	96	CC/CG	72/18	mhc class i partial
**TR15597|c0_g2_i1**	107	AA/AC	73/13	mhc class i partial
**TR15597|c0_g2_i1**	99	AA/AG	73/15	mhc class i partial
**TR15597|c0_g2_i1**	137	CC/CG	88/13	mhc class i partial
**TR15597|c0_g2_i1**	130	TT/TA	90/13	mhc class i partial
**TR15597|c0_g2_i1**	125	CC/CT	90/14	mhc class i partial
**TR15627|c1_g1_i2**	2037	CA/CC	11/15	alpha-synuclein-like
**TR15627|c1_g1_i2**	1108	AT/AA	13/16	alpha-synuclein-like
**TR15627|c1_g1_i2**	1632	TC/TT	14/11	alpha-synuclein-like
Response to stimulus (GO:0050896)				
**TR10178|c0_g1_i1**	2988	GG/GA	11/11	cystic fibrosis transmembrane conductance regulator
**TR11387|c0_g1_i1**	441	AT/AA	148/164	hypoxanthine-guanine phosphoribosyltransferase
**TR11387|c0_g1_i1**	1659	GA/GG	345/195	hypoxanthine-guanine phosphoribosyltransferase
**TR11387|c0_g1_i1**	1255	TT/TC	628/443	hypoxanthine-guanine phosphoribosyltransferase
**TR12127|c0_g2_i1**	228	GA/GG	144/70	translocation protein sec63 homolog
**TR12127|c0_g2_i1**	1634	GC/GG	300/179	translocation protein sec63 homolog
**TR12134|c0_g3_i1**	300	GA/GG	40/26	cdc42 homolog
**TR12177|c0_g1_i1**	642	CG/CC	432/285	cd59 glyco
**TR12177|c0_g1_i1**	612	AA/GG	854/381	cd59 glyco
**TR12177|c0_g1_i1**	213	GG/GA	2541/1281	cd59 glyco
**TR12177|c0_g1_i1**	309	GT/GG	3550/1779	cd59 glyco
**TR12301|c0_g2_i1**	804	TC/TT	38/33	retinoic acid receptor alpha isoform x2
**TR12301|c0_g2_i1**	210	CC/CG	41/23	retinoic acid receptor alpha isoform x2
**TR12301|c0_g2_i1**	1084	AA/AG	42/13	retinoic acid receptor alpha isoform x2
**TR13075|c0_g1_i1**	425	TT/GT	40/42	ap-2 complex subunit mu isoform x1
**TR14370|c0_g1_i1**	1650	TG/TT	12/16	adp-ribosylation factor-like protein 1
**TR14370|c0_g1_i1**	2482	CC/CG	22/17	adp-ribosylation factor-like protein 1
**TR14370|c0_g1_i1**	1788	CC/AA	26/22	adp-ribosylation factor-like protein 1
**TR14473|c0_g2_i1**	883	TT/TC	117/15	endothelial pas domain-containing protein 1

* Only 20 SNP markers for each of the three biological process were listed in this table, see S7 Table for detail information and the complete list of candidate SNP markers.

^a^: GT: genotype.

^b^: DP: read depth

## Discussion

As of September 11, 2016, only a limited number of sequences, 2,229 EST sequences, and 12 Bio-Samples with over 50 Gb sequence data were available for Eurasian perch in the NCBI SRA database. We assembled a high quality comprehensive *de novo* transcriptome for the Eurasion perch which possesses a relatively high average length of transcripts, a higher percentage (>50%) of annotated genes and more complete ORFs (Table 1 and Fig 1). The present study provides valuable genetic resources for further research on Eurasian perch.

Domestication is a long process which forces animals to adapt to captivity by modifying an animal’s growth, behavior and stress response [3, 43–45]. Generally, genes related with immune response usually under strong selection and evolve rapidly during domestication process [46]. In order to adapt to their stress responses, adaptation is often observed within the immune system, such as in the domestication of goats [47], songbirds [48], and rainbow trout [49]. During domestication, the immune response was also affected in Eurasian perch. Domestication has affected the immune responses to surgery and may lead to immune redistribution to skin in songbirds [48]. In the process of domestication of rainbow trout, the genes involved in immune function were affected [49]. Eurasian perch has also experiences changes in the immune response including changes in serum proteins related to innate/specific immunity and acute phase response [50], and differentially modulates the immune status of juvenile Eurasian perch, due to increased chronic stress by confinement [51, 52]. Many studies have supported a mechanistic link between behavior and gene expression, since changes in gene expression levels will cause phenotype variations [53, 54]. To maximize profitability, domesticated Eurasian perch were cultured in intensive farming conditions with limited space, high density, dead pond water, and other stressors, which would have led to large-scale disease [50, 51, 55]. Long-term artificial selection of individuals with high immune performance improved the survival rate of these cultured fish, which may explain the high expression of immune related genes observed in this study.

In this study, immune related genes which were highly expressed in domesticated perch included *MHCI* and *MHCII*, the L-amino-acid oxidase gene, and *ighm* (Figs 2 and 4). *MHCI* and *MHCII* are two cell surface proteins essential for the acquired immune system for antigen presentation to recognize foreign molecules in vertebrates were significantly higher expressed in domesticated Eurasian perch (Fig 4). These significantly expressed proteins would lead to the development and expansion of T cells [56]. The L-amino-acid oxidase gene displays strong antibacterial activity towards *Vibrio cholerae* and *Edwardsiella tarda* and inhibits the growth of both Gram-negative and Gram-positive bacteria [57, 58]. *Ighm* has been shown to be involved in the early recognition of bacteria, viruses and some cellular waste, which plays an important role in an organism’s primary defense mechanisms [59]. These highly expressed immune related genes identified in domesticated perch in this study may indicate a better immune performance in domesticated perch during long-term domestication. The formation of the disease resistance phenotype is a result of the interaction of certain genotypes with the intensive farming environment. Domestication has influenced both stress and immune indicators in Eurasian perch which has since improved the immune status of Eurasian carp as a response to captivity.

**Fig 5:**
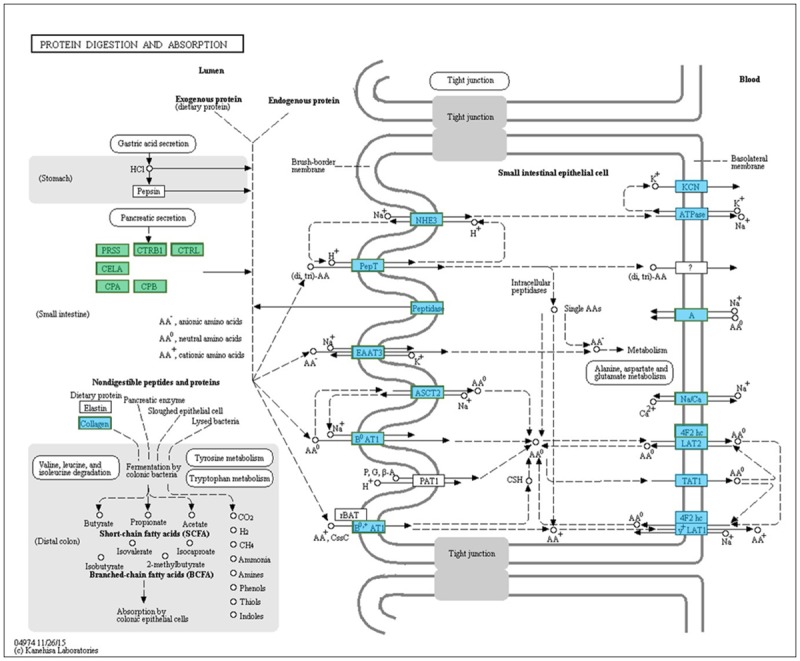
Protein digestion and absorption pathway of Eurasian perch transcriptome. Genes in blue boxes indicated these genes were identified and annotated in Eurasian perch transcriptome. Genes in green boxes indicated down-regulated genes in domesticated perch.

Long-term domestication could at some content change the digestive system of domesticates. Previous studies demonstrated that dogs have adapted to a starch-rich diet through domestication compared to the carnivorous diet of wolves [2], whereas cats developed a hypercarnivorous diet due to the positively selected genes enriched in lipid metabolism through the domestication process [28]. A total of 218 genes associated with the digestive process were down-regulated in domesticated Eurasian perch in this study. Wild Eurasian perch only accept live fish prey and refuse dead prey and artificial diets, however, domesticated Eurasian perch accept minced fillets and formula feed [60]. Natural populations of Eurasian perch compete for food and various digestive enzymes are necessary to extract as many necessary nutrients as possible from a limited food supply. On the contrary, domesticated perch were cultured in stable conditions with abundance of digestible and hyperalimentation formulated food, which may shift digestive processes. There are no longer strong selection pressures for a strong digestive ability within a captive environment. In this study, some proteolytic enzymes and some digestive related genes (*try1*, *ctr*l, *ctrb*, etc) involved in protein digestion and absorption metabolic pathway were only lowly expressed (Figs 2 and 5) in domesticated Eurasian perch, may be the result of a captive diet. These captive fish are no longer under strong selection pressure to maximize absorption of limited resources, such as protein, so although these enzymes and metabolic pathways may be more effective in protein digestion, they are less active in domesticated perch [15, 51].

This study supplied a comprehensive transcriptome for Eurasian perch as well as annotated genes and potential SNPs which may support future studies and aquaculture efforts for Eurasian perch. Furthermore, this study also revealed that domestication has altered the immune and digestive systems of Eurasian perch. By up-regulating genes involved in the immune response fish may be more capable of responding to increased stress caused by confinement. By down-regulating proteolytic enzyme and metabolic pathways for protein absorption, captive Eurasian perch are able to reduce the uncessary energy expended for digestion because they are supplied with an unlimited food source.

## Data archiving

Sequencing reads are available at NCBI SRA database (SRX2317605).

## Supporting information

S1 Fig Species distribution of top-blast hits in the assembled reference transcriptome. (PDF)

S2 Fig Functional classification of the Eurasian perch transcriptome based on three main Gene Ontology (GO) categories. (PDF)

S3 Fig The distribution of the putative SNPs associated with growth, immune system process, and response to stimulus. (PDF)

S1 Table Information of qRT-PCR primers for 16 selected genes. (PDF)

S2 Table Total number of reads, and mapping stastics of domesticated and wild Eurasian perch. (PDF)

S3 Table Distribution of percent length coverage for the top matching uniprot database entries. (PDF)

S4 Table KEGG pathways with numbers of transcripts and enzymes from transcriptomic analysis of Eurasian perch. (XLSX)

S5 Table Prediction results of protein domains. (XLSX)

S6 Table Putative SNP markers identified to distinguish domesticated and wild perch. (XLSX)

S7 Table Putative SNP markers identified to distinguish domesticated and wild perch, related to growth (GO:0040007), immune system process (GO:0002376), and response to stimulus (GO:0050896) from the reference transcriptome. (XLSX)
