# Supplementary figures and images for: Domestication drive the changes of immune and digestive system of Eurasian perch (Perca fluviatilis)
Source: PLoS One. 2017 Mar 3;12(3):e0172903. doi: 10.1371/journal.pone.0172903 (PMC5336236; doi:10.1371/journal.pone.0172903)

S1 Fig. Species distribution of top-blast hits in the assembled reference transcriptome.

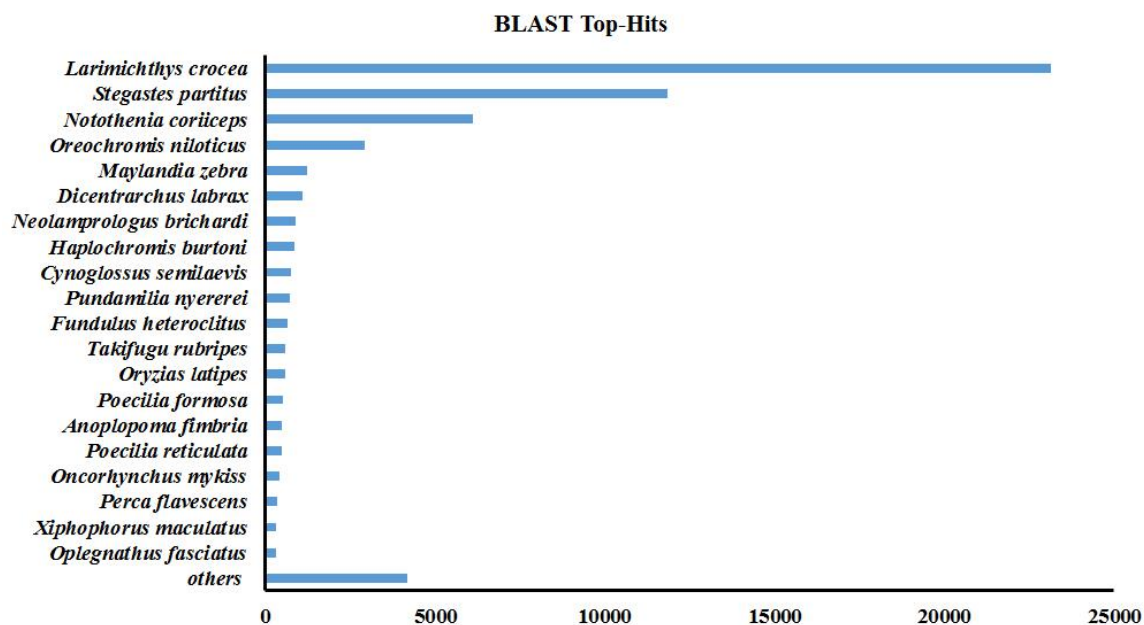

Supplement: S1 Fig — (PDF) [file pone.0172903.s001.pdf]
