# Supplementary material for: Domestication drive the changes of immune and digestive system of Eurasian perch (Perca fluviatilis)
Source: PLoS One. 2017 Mar 3;12(3):e0172903. doi: 10.1371/journal.pone.0172903 (PMC5336236; doi:10.1371/journal.pone.0172903)

S2 Fig. Functional classification of the Eurasian perch transcriptome based on three main Gene Ontology (GO) categories.

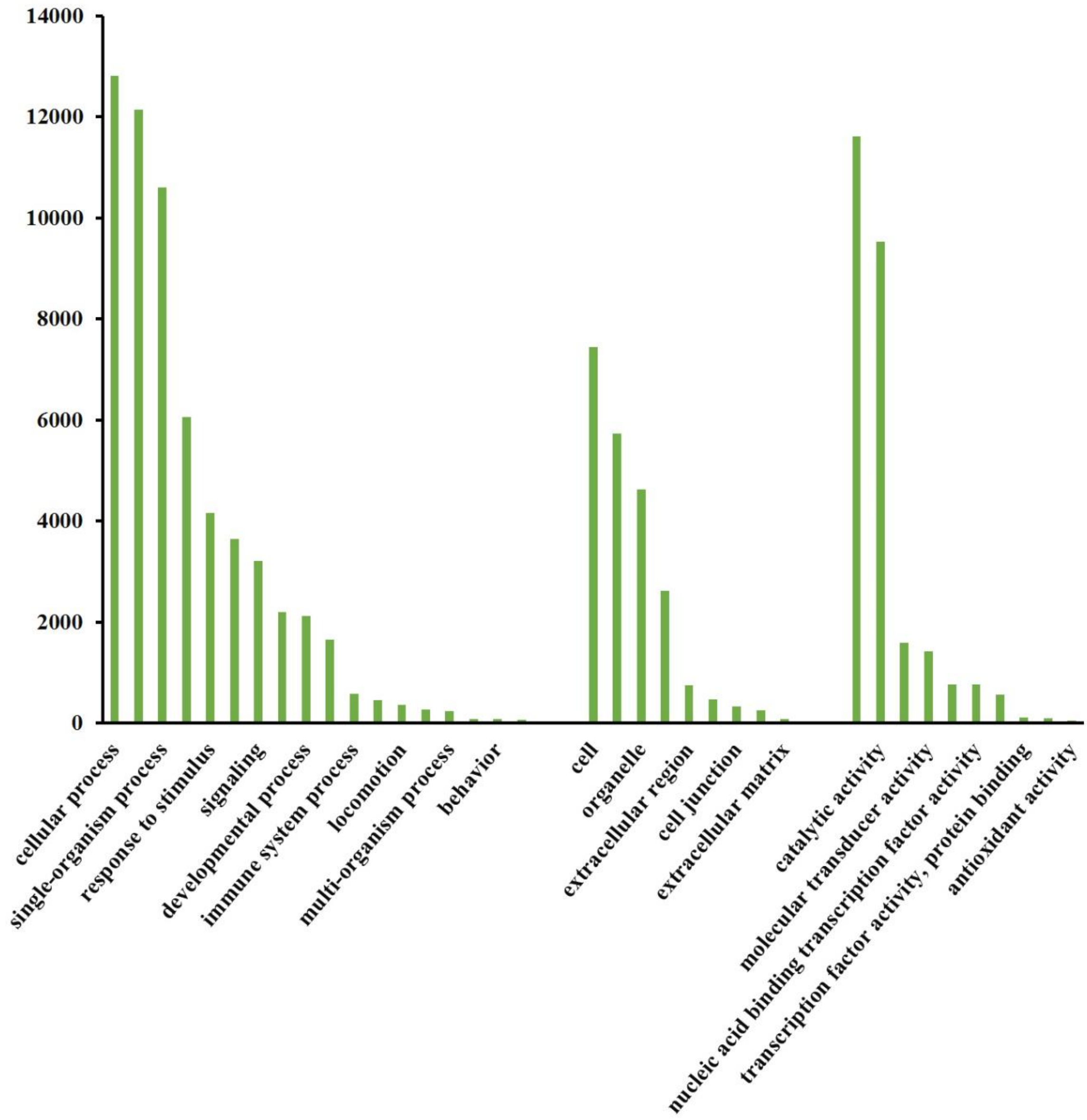

Supplement: S2 Fig — (PDF) [file pone.0172903.s002.pdf]
