# Supplementary material for: Domestication drive the changes of immune and digestive system of Eurasian perch (Perca fluviatilis)
Source: PLoS One. 2017 Mar 3;12(3):e0172903. doi: 10.1371/journal.pone.0172903 (PMC5336236; doi:10.1371/journal.pone.0172903)

S3 Fig. The distribution of the putative SNPs associated with growth, immune system process, and response to stimulus.

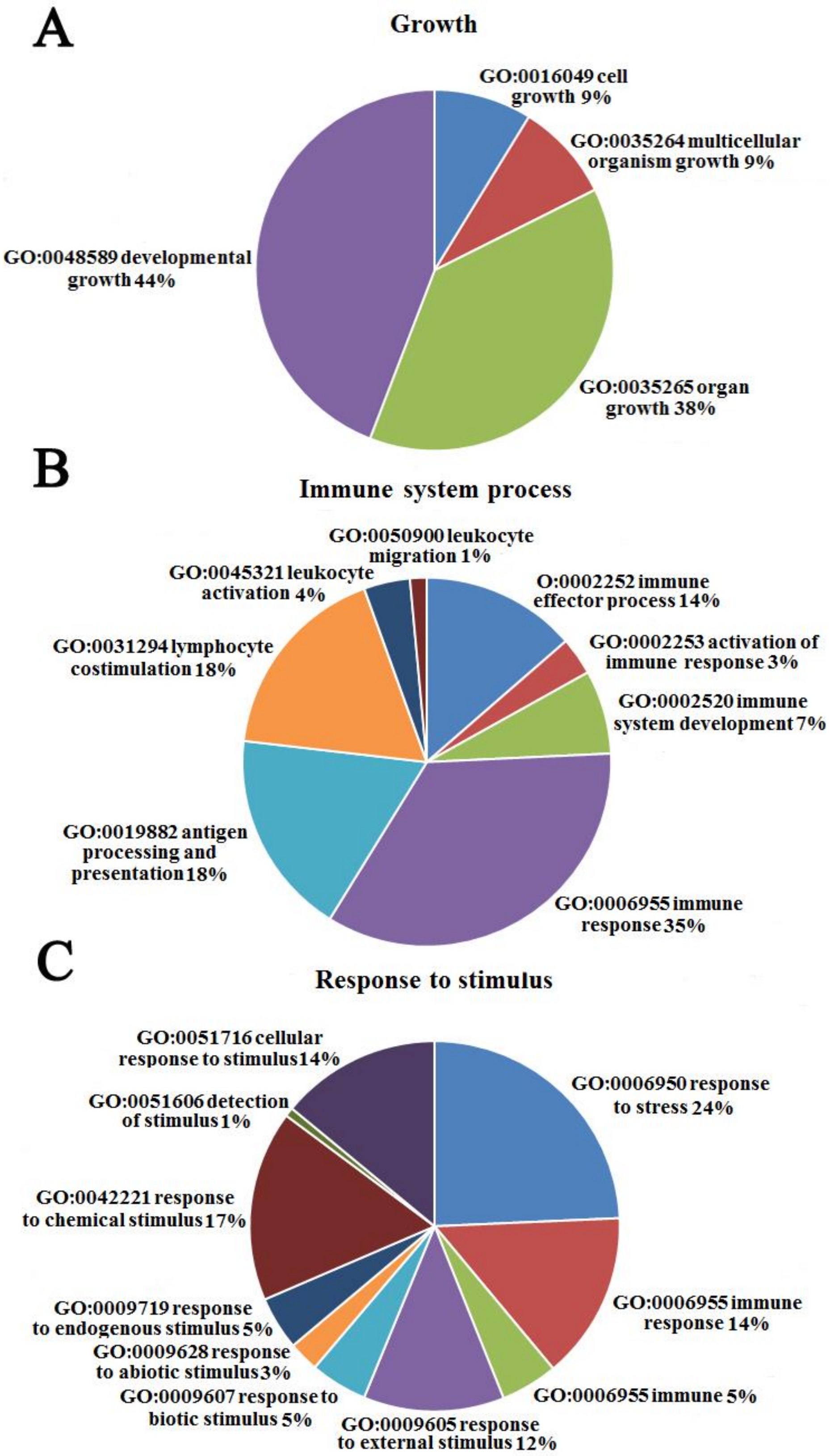

Supplement: S3 Fig — (PDF) [file pone.0172903.s003.pdf]
