# Supplementary material for: Domestication drive the changes of immune and digestive system of Eurasian perch (Perca fluviatilis)
Source: PLoS One. 2017 Mar 3;12(3):e0172903. doi: 10.1371/journal.pone.0172903 (PMC5336236; doi:10.1371/journal.pone.0172903)
Supplement: S1 Table — (PDF) [file pone.0172903.s004.pdf]

S1 Table. Information of qRT-PCR primers for 16 selected genes

**Primer**	**Sequence (5'-3')**
β-actin	F: AGGCTGTGCTGTCCCTGTAT
	R: CATAGATGGGCACTGTGTGG
cel2a-1	F: TCCAGCCACGATTCCATCCC
	R: CATGCCACCTGCTCCCAGTC
cel2a-2	F: GCAGCGTCTCGTAAGGGTAG
	R: GATTGATGTCCATCCTGGCT
ctrb	F: GTGTCTCTGCAGCAATCCAA
	R: GATCACACGGTGGTACGTCA
cel3b	F: CCTTGCTGCCTGTGGTGGAG
	R: CAGTTGAGAGGACCGCCAGAG
ctrl	F: GCAGGTAGGACACACGTGAA
	R: GGTCCCTTGTGGGTATTGTG
isk	F: AGCGATGGAAACACCTATGC
	R: CAGCTTTCCTCTTTGGCAAC
tyr1	F: CCACAGAAGTGGTAGCCAGAG
	R: CCACGGAGGATGACAAGATT
tyr3	F: CATTGTCCAGGTTGTTGCTG
	R: ACTGCTACAAGTCCCGCATC
hb23	F: TGTTGGTCTGCAGCGTCTAC
	R: GCCAGCTCATCAGTGGAAGT
ighm	F: CGGCTTCACACCCTCCTCAC
	R: AAATCTCCCTTGCGTCCCAGTC
chia-1	F: ACTTCTGTCTGTTGGCTGGAGAG
	R: TGCTGGCTGTTGGTGGATGG
chia-2	F: GGGTCGTGCTGCCTTCATCA
	R: GCTGCCGTTGTGTCCAGGAT
mep1b	F: TGGAGGATGAGGATGAGGAG
	R: GCTCATTGGCTTCCATGAAT
MHC Ⅰ	F: TGGCTCTGTCAAGGTAGAAACCTGT
	R: TGGGAGGAGCTCTTTGCTGAAC
hmr1-a	F: GAGTCCCAAACTTCCCAGAG
	R: TTCTTTGGCTCTGCTCATCC
hmr1-b	F: GAGCTGGTAGGACGGTGTGT
	R: CAAGCCAGGTCTTCATTGTG
