# Supplementary material for: Domestication drive the changes of immune and digestive system of Eurasian perch (Perca fluviatilis)
Source: PLoS One. 2017 Mar 3;12(3):e0172903. doi: 10.1371/journal.pone.0172903 (PMC5336236; doi:10.1371/journal.pone.0172903)
Supplement: S2 Table — (PDF) [file pone.0172903.s005.pdf]

S2 Table. Total number of reads, and mapping stastics of domesticated and wild Eurasian perch

	No. of reads	No. of mapped reads	No. of properly paried mapped reads	mapping ratio
Domesticated group	90743935	89171760	80847325	98.27%
Wild group	77157339	76278521	69806762	98.86%
Total	167901388	165450415	150654675	98.54%
