# Supplementary material for: Domestication drive the changes of immune and digestive system of Eurasian perch (Perca fluviatilis)
Source: PLoS One. 2017 Mar 3;12(3):e0172903. doi: 10.1371/journal.pone.0172903 (PMC5336236; doi:10.1371/journal.pone.0172903)
Supplement: S3 Table — (PDF) [file pone.0172903.s006.pdf]

S3 Table. Distribution of percent length coverage for the top matching uniprot database entries

#hit_pct_cov_bin	count_in_bin	>bin_below
100	7472	7472
90	1798	9270
80	1322	10592
70	1210	11802
60	1328	13130
50	1338	14468
40	1406	15874
30	1421	17295
20	1517	18812
